# The relationship between athletes’ music in mood regulation and positive psychological capital and athletic performance

**DOI:** 10.3389/fpsyg.2025.1509535

**Published:** 2025-10-01

**Authors:** Young-Jun Choi, Han-Byul Kim, Kyungjin Kim

**Affiliations:** ^1^Department of Sports and Leisure Studies, Busan University of Foreign Studies, Busan, Republic of Korea; ^2^Department of Physical Education, Korea National Sport University, Seoul, Republic of Korea; ^3^Department of Adapted Physical Education, Korea National Sport University, Seoul, Republic of Korea

**Keywords:** athletes, music in mood regulation, positive psychological capital, athletic performance, South Korea

## Abstract

**Introduction:**

This study explores the connection between athletes’ use of music in mood regulation, positive psychological capital, and athletic performance. While music is widely acknowledged for its psychological benefits, its role in enhancing athletes’ posi-tive psychological capital and influencing performance outcomes remains insufficiently understood.

**Methods:**

A survey was conducted with 417 athletes, and the collected data were ana-lyzed using descriptive statistics, confirmatory factor analysis (CFA), correlation analysis, and structural equation modeling (SEM) to examine the research content.

**Results:**

The results showed that, first, athletes’ use of music for mood regulation had a positive effect on the positive psychological capital. Second, athletes’ use of music for mood regulation did not have a statistically significant effect on athletic performance. Third, the positive psychological capital of athletes had a positive effect on the athletic performance.

**Discussion:**

It is necessary to provide education and strategies that help athletes regulate their mood using music. Further, the research on the effects of music in mood regulation, which can be expected to positively affect athletes’ positive psychological capital and athletic performance, should be expanded.

## 1 Introduction

Why do humans listen to music? Scholars from various academic disciplines offer diverse answers to this question. Generally, music listening is known to be beneficial for an individual’s psychological and physiological functions (Thoma et al., 2012). It was reported to be an effective medium for affirming one’s identity, expressing, or regulating emotions (Campbell et al., 2007; North et al., 2000; Saarikallio, 2012). Additionally, it helps reduce negative emotions such as depression, anxiety, and pain, and aids in achieving emotional comfort and stability through hormonal and brainwave changes (Brennan and Charnetski, 2000). Music listening, therefore, is inseparable from human life and culture.

Music listening has been widely recognized as an effective means of mood regulation through several studies (North et al., 2000; Saarikallio, 2012). In particular, music listening functions as a mechanism for emotion regulation and stress relief, with various musical elements—such as genre, volume, and beats per minute (BPM)—having a direct impact on mood regulation (Miranda and Claes, 2009; Juslin and Sloboda, 2010). For instance, slow-tempo music (60–80 BPM) has been reported to promote relaxation and a sense of calm, whereas fast-tempo music (120 BPM or higher) increases arousal levels and energy (Thoma et al., 2013). Additionally, lyrical and classical music has been found to aid in stress reduction and enhanced concentration, while intense music genres such as rock and metal can induce emotional arousal and excitement (Labbé et al., 2007). Thus, the effects of music listening on mood regulation are influenced not only by an individual’s musical preferences but also by the specific characteristics of the music and the psychological context in which it is experienced.

Furthermore, recent research has empirically validated the mood-regulating effects of music listening through various interdisciplinary approaches (Gibbs and Egermann, 2021; Henry et al., 2021). Research suggests that music-based mood regulation strategies can be broadly categorized into emotional healing, mood enhancement, and emotional release (Saarikallio and Erkkilä, 2007). Specifically, in terms of emotional healing, classical music and nature-based sounds have been found to be effective in alleviating negative emotions and promoting psychological relaxation (Brennan and Charnetski, 2000). Regarding mood enhancement, music with bright melodies and a moderate tempo (80–120 BPM), particularly pop music, has been shown to be most effective in reinforcing positive emotions (Chin and Richard, 2012). In the domain of emotional release, intense music genres such as rock and metal facilitate the expression of negative emotions and, under certain psychological conditions, contribute to stress relief (Van den Tol and Edwards, 2013). According to previous research (Gibbs and Egermann, 2021; Henry et al., 2021; Wininger and Pargman, 2003), people use music to change or regulate their mood. Music listening helps alleviate negative emotions, thereby contributing to psychological healing and recovery. It provides pleasure and self-satisfaction in the emotional realm. Music listening plays a role in enhancing positive emotional states and alleviating negative ones (Chin and Richard, 2012).

Listening to music for mood regulation is more closely related to positive states rather than negative ones and is observed across various age groups (Laukka, 2007; Morinville et al., 2013). Saarikallio and Erkkilä (2007) analyzed interview content conducted with adolescents and proposed a theoretical model explaining mood regulation as a process of improving or maintaining one’s mood state through various music activities. They developed the Music in Mood Regulation Scale (MMR) to evaluate how music is used to regulate mood and its effectiveness. Since the MMR was based on the musical experiences of adolescents, it can provide important insights into understanding the self-regulatory behavior of younger generations. Further, it can measure the psychological effects and patterns of music on everyday mood states (Lee, 2015).

Specifically, a study (Lee and Kim, 2019) found that using music to regulate mood was more closely related to enhancing positive emotions than alleviating negative ones. It was more strongly associated with emotional states while listening to music rather than typical emotional states. Such findings indicate that music in mood regulation has a significant impact on positive psychological resources, or positive psychological capital, as demonstrated by various studies (Thoma et al., 2012; Bradt and Dileo, 2014; Chan et al., 2012; Juslin and Västfjäll, 2008).

The positive psychological capital is the ability to positively draw out one’s potential in everyday or challenging situations, providing a positive psychological state that allows for adaptation to the given environment and successful achievement of set goals (Luthans et al., 2007a). Luthans (2002) introduced the concept of positive psychological capital, integrating four components: self-efficacy, hope, resilience, and optimism (Luthans et al., 2007a; Luthans and Youssef, 2004).

In addition, the positive psychological capital has been defined as an individual’s positive cognitive state for achievement and success (Luthans et al., 2007b), and it is considered an important resource for enhancing the performance of both individuals and organizations. The positive effects of positive psychological capital on individuals and organizations (Batool et al., 2023; Kim and Hong, 2023; Moon and Jung, 2022) include providing confidence to overcome challenges with a positive attitude (Moon and Jung, 2022), promoting innovative behavior that improves performance (Kim and Hong, 2023), and strengthening the will to contribute to shared goals and success (Batool et al., 2023). It contributes to an individual’s positive psychological state and the ability to achieve success in various environments.

A group of participants in the research where positive psychological capital is highly required is athletes. Athletes experience high levels of physical and mental stress as they need not only physical training but also mental preparation to achieve high performance in competitions. The positive psychological capital plays a critical role in improving athletes’ performance and enhancing mental resilience, as it helps them overcome stress and pressure and provides continuous motivation. Gabana (2019) reported that a positive psychological state significantly contributes to the improvement of athletes’ performance, suggesting a strong correlation between the positive psychological capital and performance.

What is the most important goal for athletes? The answer is performance. The development and improvement of performance are crucial factors for progressing to higher levels of education, such as advancing to a higher school, and significantly impact career opportunities, such as joining professional teams (Choi, 2022). Moreover, the performance is of para-mount importance in South Korea’s sports environment, where the “win at all costs” mentality prevails. However, measuring performance often refers to actual competition results, which can be difficult to collect. Athletic performance is a complex concept influenced by physiological, psychological, biomechanical, and sports science-related factors that interact with each other.

Athletic performance is a complex concept influenced by physiological, psychological, biomechanical, and sports science-related factors that interact with each other. Direct measurements of physiological or performance-related data, such as maximal oxygen uptake (VO_2_max), anaerobic threshold, muscular strength and endurance, neuromuscular control, and time to exhaustion, represent objective direct measurement methods for assessing physical performance.

On the other hand, indirect measurement methods, which utilize self-report measures (e.g., questionnaires) and coach evaluations, assess psychological readiness factors such as motivation, confidence, and concentration. These psychological constructs are grounded in well-established theories, including self-efficacy theory (Bandura, 1977), flow theory (Csikszentmihalyi, 1990), goal setting, imagery training, and routine application, which are essential components of psychological skills training (PST).

Athletic performance assessment has been examined from multiple academic perspectives. Physical performance alone does not fully explain athletic performance, as psychological, cognitive, and environmental factors play a crucial role. Self-report measures provide a valuable tool for quantitatively evaluating these multidimensional components, and their validity has been substantiated through various studies. Specifically, Gould et al. (1999) demonstrated that athletic performance is strongly associated with psychological factors such as confidence, motivation, anxiety, and concentration, which can be effectively measured through self-report questionnaires. Similarly, Weinberg and Gould (2018) emphasized the importance of psychological assessments in predicting athletic performance.

This study also employs self-report measures to quantitatively analyze athletes’ cognitive, psychological, and environmental factors using a validated questionnaire based on previous research. The selected questionnaire has been tested for reliability and validity, making it a suitable method for evaluating the relationship between psychological factors and athletic performance. Consequently, self-report measures can serve as a valuable tool for integrating subjective experiences with quantitative data to provide a more comprehensive assessment of athletic performance.

For example, the Rating of Perceived Exertion (RPE) scale is widely recognized as a reliable method for assessing subjective exercise intensity and fatigue perception, with well-established validity in its relationship with athletic performance. Therefore, in order to ensure the reliability and validity of athletic performance assessments, it is logically appropriate to incorporate performance execution as a key measurement variable, considering athletes’ psychological and cognitive factors in a comprehensive analysis.

The athletic performance refers to the ability of an individual or team to perform in a competition or the comprehensive ability encompassing the various skills exhibited by individual athletes or teams (Kim, 2019). The goal of elite sports is optimal athletic performance, and to achieve this, both athletes and coaches devote significant effort in training and other areas (Kim and Lee, 2020). Given its importance and its culmination in the goal of athletes -athletic performance- it has been studied as a dependent variable in relation to various other variables. While studies have investigated the relationship between psycho-logical factors such as competitive spirit (Kim and Lee, 2020; Kim and Ahn, 2020), sports immersion (Song, 2021), confidence (Park, 2019), and passion (Nam et al., 2023), no research has yet explored the relationship between music in mood regulation and athletic performance.

This study aims to analyze the relationship between music in mood regulation and various variables in athletes—a subject that has not been explored before—and holds significance as the first attempt in the field of sports. The results are expected to provide valuable information to coaches and athletes in the field of sports coaching.

Therefore, the purpose of this study was to empirically analyze the relationship between music in mood regulation, positive psychological capital, and athletic performance in athletes. The research hypotheses established to achieve the study’s purpose are as follows:

Hypothesis 1: Music-based mood regulation in athletes will be related to positive psychological capital.

Hypothesis 2: Music-based mood regulation in athletes will be related to athletic performance.

Hypothesis 3: Positive psychological capital in athletes will be related to athletic performance.

## 2 Methods

### 2.1 Participants

The participants of the study consisted of 417 athletes registered with the Korea Sports Council and affiliated with Universities A and B, selected using a convenience sampling method. After explaining the purpose and content of the study to the coaches and athletes of each university’s sports teams and obtaining their cooperation, data were collected via an online survey using Naver Forms. The demographic characteristics of the participants are presented in Table 1.

**Table 1:** Demographic characteristics of participants in the study.

Characteristics		Frequency (*N*)	Percentage (*%*)
Gender	Male	209	50.1
	Female	208	49.9
Award winning experience	None	31	7.4
	Winning a municipal competition	22	5.3
	A national convention prize	297	71.2
	An international prize	67	16.1
Athletic experience	3–6 years	89	21.3
	6–9 years	151	36.2
	9–12 years	110	26.4
	12–15 years	43	10.3
	More than 15 years	24	5.8

Although the optimal sample size, calculated based on the national athlete population (357,627 registered athletes as of July 2024) at a 95% confidence level with a 5% margin of error, would be approximately 384, we initially recruited 450 participants. After excluding incomplete or invalid responses, 417 valid cases were retained for analysis. This approach considered potential missing data, dropout rates (~10%), and practical constraints, and aligns with widely accepted standards in applied research regarding sample adequacy and methodological rigor.

### 2.2 Research instrument

#### 2.2.1 Music in mood regulation

The Music in Mood Regulation scale used in this study was an adapted and validated Korean version of the MMR originally developed by Saarikallio, (2008), which was translated and validated by Lee (2015). This modified and supplemented scale, known as the K-MMR, was used to measure music in mood regulation. The sub-factors of music in mood regulation included 18 items categorized into six dimensions: revival (3 items), discharge (3 items), strong sensation (3 items), entertainment (3 items), diversion (3 items), and solace (3 items). Each item was measured on a 5-point Likert scale ranging from 1 (strongly disagree) to 5 (strongly agree).

#### 2.2.2 Positive psychological capital

The Positive Psychological Capital (PsyCap) was measured using the Korean version of the Positive Psychological Capital Scale (K-PPC), which was originally developed by Luthans et al. (2007a) (Chan et al., 2012) and later translated and validated for cross-cultural use by Lim (2014). The K-PPC scale consists of 18 items across four sub-dimensions: self-efficacy (5 items), optimism (5 items), hope (5 items), and resilience (3 items). Each item was measured on a 5-point Likert scale, ranging from 1 (strongly disagree) to 5 (strongly agree).

#### 2.2.3 Athletic performance

Athletic Performance was measured using a questionnaire developed by Kim (2017), which was adapted and supplemented from the scales originally developed by Duda and Nicholls (1992) and Hersey and Blanchard’s (1969) subordinate evaluation scale, which was translated and used by Kim (1995). The Athletic Performance scale consisted of 16 items divided into three sub-factors: match performance success (7 items), psychological maturity (5 items), and match performance maturity (4 items). Each item was measured on a 5-point Likert scale, ranging from 1 (strongly disagree) to 5 (strongly agree).

### 2.3 Validity and reliability of research instrument

To validate the research instrument, Confirmatory Factor Analysis (CFA) was conducted using the Maximum Likelihood (ML) method. The model fit was assessed using the χ^2^ test, SRMR (standardized RMR), RMSEA (root mean square error of approximation), CFI (comparative fit index), and TLI (Tucker-Lewis index). The criteria for model fit are as follows: the significance value of the χ^2^ test should be greater than 0.05, SRMR should be 0.08 or below, RMSEA should be 0.10 or below, and CFI and TLI should be 0.90 or above for the model to be considered a good fit (Duda and Nicholls, 1992; Hersey and Blanchard, 1969). Additionally, the reliability was verified using Cronbach’s *α* coefficient.

The results of the CFA indicated that the model fit indices were χ^2^(df)/*p* = 2629.261(1196)/0.001, SRMR = 0.045, RMSEA = 0.054, CFI = 0.916, and TLI = 0.907, which meet the criteria for model fit. The Composite Reliability (CR) ranged from 0.829 to 0.934, and the Average Variance Extracted (AVE) ranged from 0.564 to 0.836. The reliability coefficients ranged from 0.821 to 0.836. The results of the validity and reliability were specified in Table 2.

**Table 2:** The results of the validity and reliability.

Variable	Question	Non-standardization coefficient	Standardization coefficient	SE	*t*	Degree of significance	Reliability	CR	AVE
Revival	m1	1.000	0.933				0.934	0.939	0.836
	m2	1.026	0.936	0.030	34.196	***			
	m3	0.966	0.866	0.035	27.822	***			
Discharge	m4	1.000	0.917				0.909	0.858	0.668
	m5	0.995	0.910	0.037	27.064	***			
	m6	0.895	0.811	0.040	22.203	***			
Strong sensation	m7	1.000	0.846				0.875	0.840	0.636
	m8	0.960	0.812	0.049	19.415	***			
	m9	1.102	0.852	0.053	20.753	***			
Entertainment	m10	1.000	0.700				0.821	0.829	0.618
	m11	1.338	0.814	0.091	14.641	***			
	m12	1.305	0.832	0.088	14.859	***			
Diversion	m13	1.000	0.833				0.901	0.887	0.724
	m14	1.167	0.915	0.049	23.689	***			
	m15	1.065	0.864	0.049	21.749	***			
Solace	m16	1.000	0.901				0.893	0.874	0.698
	m17	1.034	0.890	0.040	26.030	***			
	m18	0.845	0.791	0.041	20.870	***			
Self-efficacy	p1	1.000	0.804				0.927	0.943	0.767
	p2	1.059	0.838	0.053	19.895	***			
	p3	1.018	0.868	0.049	20.952	***			
	p4	1.037	0.874	0.049	21.170	***			
	p5	1.039	0.848	0.051	20.259	***			
Optimism	p6	1.000	0.708				0.852	0.874	0.582
	p7	0.873	0.653	0.069	12.593	***			
	p8	1.026	0.775	0.069	14.869	***			
	p9	0.916	0.712	0.067	13.708	***			
	p10	1.087	0.816	0.070	15.623	***			
Hope	p11	1.000	0.770				0.892	0.912	0.674
	p12	1.026	0.806	0.058	17.721	***			
	p13	1.031	0.729	0.066	15.715	***			
	p14	1.075	0.852	0.057	18.978	***			
	p15	1.098	0.795	0.063	17.432	***			
Resilience	p16	1.000	0.824				0.889	0.878	0.705
	p17	1.139	0.866	0.055	20.553	***			
	p18	1.061	0.872	0.051	20.705	***			
Match performance success	gp1	1.000	0.852				0.888	0.911	0.597
	gp2	0.963	0.826	0.046	21.000	***			
	gp3	0.847	0.780	0.044	19.160	***			
	gp4	0.845	0.768	0.045	18.727	***			
	gp5	0.949	0.830	0.045	21.170	***			
	gp6	0.732	0.616	0.061	11.983	***			
	gp7	0.787	0.638	0.063	12.466	***			
Psychological maturity	gp8	1.000	0.803				0.810	0.865	0.564
	gp9	0.838	0.589	0.077	10.941	***			
	gp10	1.148	0.740	0.089	12.951	***			
	gp11	0.986	0.810	0.096	10.275	***			
	gp12	1.118	0.827	0.091	12.240	***			
Match performance maturity	gp13	1.000	0.758				0.868	0.841	0.576
	gp14	0.895	0.774	0.047	19.102	***			
	gp15	1.002	0.551	0.059	17.006	***			
	gp16	0.944	0.569	0.054	17.491	***			

*χ*^2^ = 2629.261, *df* = 1,196, ***p = 0.001, SRMR = 0.045, RMSEA = 0.054, CFI = 0.916, TLI = 0.907.

### 2.4 Data analysis

Statistical analysis was conducted using the SPSS 29.0 and AMOS 29.0 statistical programs. The statistical significance level was set at 0.05. The demographic characteristics of the participants were analyzed using frequency analysis and descriptive statistics. The validity of the research instrument was verified through the CFA by examining factor loadings and model fit and calculating the AVE and CR. The reliability was assessed by calculating Cronbach’s α coefficient. To analyze the relationships between variables according to the research content, correlation analysis and structural equation modeling (SEM) were conducted.

## 3 Results

### 3.1 Results of the correlation analysis

The results of the correlation analysis were presented in Table 3. The analysis revealed that there is a statistically significant positive correlation between music in mood regulation, positive psychological capital, and athletic performance. The correlation coefficients ranged from 0.172 to 0.781, indicating that they do not exceed the threshold of 0.850 suggested by Kline (2011). Therefore, it was confirmed that there is no issue of multicollinearity in the data.

**Table 3:** Results of the correlation analysis.

	1	2	3	4	5	6	7	8	9	10	11	12	13
1	1												
2	0.321***	1											
3	0.593***	0.480***	1										
4	0.622***	0.271***	0.552***	1									
5	0.703***	0.397***	0.637***	0.640***	1								
6	0.607***	0.446***	0.706***	0.576***	0.735***	1							
7	0.388***	0.276***	0.388***	0.413***	0.426***	0.409***	1						
8	0.361***	0.228***	0.363***	0.394***	0.376***	0.371***	0.749***	1					
9	0.347***	0.172***	0.344***	0.443***	0.411***	0.392***	0.781***	0.755***	1				
10	0.256***	0.199***	0.240***	0.294***	0.235***	0.255***	0.633***	0.620***	0.666***	1			
11	0.322***	0.244***	0.400***	0.419***	0.411***	0.412***	0.699***	0.687***	0.728***	0.588***	1		
12	0.262***	0.191***	0.279***	0.354***	0.344***	0.287***	0.705***	0.659***	0.732***	0.546***	0.698***	1	
13	0.342***	0.200***	0.338***	0.413***	0.393***	0.378***	0.700***	0.697***	0.772***	0.585***	0.721***	0.698***	1

*p < 0.05, **p < 0.01, ***p < 0.001. 1. Revival, 2. Discharge, 3. Strong sensation, 4. Entertainment, 5. Diversion, 6. Solace, 7. Self-efficacy, 8. Optimism, 9. Hope, 10. Resilience, 11. Match performance success, 12. Psychological maturity, 13. Match performance maturity.

### 3.2 Results of the structural model conformity

The results of fit indices for the structural model were shown in Table 4. The indices were as follows: *χ*^2^(*df*) = 154.325(62)/*p* < 0.001, SRMR = 0.038, RMSEA = 0.060, CFI = 0.976, and TLI = 0.970, indicating that the fit indices meet the acceptable criteria.

**Table 4:** Results of the structural model conformity.

Structural model conformity	*χ^2^*	*df*	*p*	SRMR	RMSEA	CFI	TLI
	154.325	62	0.001	0.038	0.060	0.976	0.970

### 3.3 Results of the hypothesis verification

The results of the analysis on the relationship between athletes’ music in mood regulation, positive psychological capital, and athletic performance are presented in Table 5. First, the analysis of the relationship between athletes’ music in mood regulation and positive psychological capital showed a standardized coefficient value of 0.534 (*t* = 10.389, *p* = 0.001), indicating that music in mood regulation had a statistically significant positive effect on positive psychological capital. Second, the analysis of the relationship between athletes’ music in mood regulation and athletic performance showed a standardized coefficient value of 0.019 (*t* = 0.549, *p* = 0.583), indicating that music in mood regulation did not have a statistically significant effect on athletic performance. Third, the analysis of the relationship between athletes’ positive psychological capital and athletic performance showed a standardized coefficient value of 0.956 (*t* = 19.447, *p* = 0.001), indicating that positive psychological capital had a statistically significant positive effect on athletic performance.

**Table 5:** Results of the hypothesis verification.

Path	Path Coefficient (Standardization coefficient)	SE	C.R (*p*)	Result
Music in mood regulation → Positive psychological capital	0.416(0.534)	0.040	10.389 (0.001)	Acceptance
Music in mood regulation → Athletic performance	0.013(0.019)	0.024	0.549 (0.583)	Rejection
Positive psychological capital → Athletic performance	0.856(0.956)	0.044	19.447 (0.001)	Acceptance

## 4 Discussion

This study aimed to explore the relationship between athletes’ music in mood regulation, positive psychological capital, and athletic performance. Based on the research findings, the following discussion is presented.

First, it was found that athletes’ music in mood regulation positively influences the positive psychological capital. This result aligns with the findings of Lee and Kim (2019), who reported that music in mood regulation positively impacts positive psychology. Music listening is utilized as a tool that positively affects emotional regulation, playing a role in reducing stress, overcoming depression, and promoting relaxation (Juslin and Laukka, 2004; Krout, 2007; Miranda and Claes, 2009). Further, the results support the notion that music listening enhances positive emotional states and alleviates negative ones (Chin and Richard, 2012).

Music listening is known as an effective means for regulating emotions and mood (North et al., 2000; Pang, 2022). These findings suggest that music serves not only as a temporary mood-enhancing tool but also as a mechanism for accumulating psychological resources over the long term. Through repeated engagement with specific types of music, athletes may develop an association between music and positive emotional states, which could, in turn, contribute to long-term psychological resilience and the reinforcement of self-regulation strategies (Koelsch, 2014). Therefore, further research is needed to examine whether music-based mood regulation strategies not only facilitate short-term emotional adjustments but also contribute to the sustained development of positive psychological capital. The results suggest that music in mood regulation helps athletes achieve psychological stability, enhance positive emotions, and thereby strengthen the components of positive psychological capital, such as hope and optimism. According to Stevens and Lane (2001), athletes use various mood regulation strategies, with music playing an important role. This indicates that the belief and feeling that athletes can regulate and im-prove their mood may enhance their self-efficacy, which positively influences positive psychological capital. These findings suggest that music functions not only as a tool for emotional regulation but also as a means to enhance athletes’ self-regulation capacity and psychological control. In particular, athletes experience significant psychological pressure not only before and after competitions but also throughout their training process (Hall et al., 2021), and this study confirms that music can serve as a strategy to manage such pressures. Future research should explore the interaction between individual athlete characteristics (e.g., personality traits, emotional sensitivity) and the effects of music listening, thereby developing more personalized mood regulation strategies.

Ellingson (2003) reported that athletes use music to prepare psychologically for competitions. The use of music for psychological preparation likely contributes to enhancing the positive psychological capital, including self-efficacy, optimism, hope, and resilience, which are beneficial for performance. However, the effects of music are not solely determined by its presence but also by the context in which it is used. For instance, the psychological functions of music during training and pre-competition phases may differ significantly (Karageorghis and Terry, 2011). While music during training can enhance physical endurance and motivation, its primary role in the pre-competition phase may be to promote psychological stability and maintain focus (Bishop et al., 2008). Therefore, further research is needed to examine how athletes utilize music at different time points and how this influences their positive psychological capital and athletic performance. Additionally, the study noted that athletes use various artists and tracks when preparing for competitions, suggesting the need for further research into the nuances of music in mood regulation. While this study analyzed the relationship between music in mood regulation and positive psychological capital, future research could provide valuable practical insights by exploring the types and timing of music used by athletes during mood regulation.

Terry and Karageorghis (2006) found that music’s melody and harmony positively change athletes’ moods. They reported that listening to music associated with positive past experiences can increase motivation and induce positive emotions. This suggests that music in mood regulation helps athletes set and achieve goals, thereby boosting the hope and optimism components of positive psychological capital. Thus, it is recommended that coaches incorporate music into training to help athletes have the positive experiences while performing. More specifically, these findings highlight the need for a more systematic integration of music in sports coaching and competition preparation programs. Coaches can assist athletes in maintaining an optimal psychological state by curating personalized music playlists that align with individual psychological characteristics and the specific demands of their sport (Bishop et al., 2008). Additionally, incorporating Psychological Skills Training (PTS) through music could serve as an effective strategy, enabling athletes to autonomously regulate their psychological state in high-stress situations (Terry and Karageorghis, 2006).

Moreover, the music is not only immediate but also conveys specific emotions in-tensely and swiftly (Robinson, 2005). Strategies for mood regulation can occur at sensory, cognitive, and behavioral levels, or through interactions with the environment, at various levels (Campos et al., 2004). Athletes tend to synchronize their movements to the rhythm of music, which can increase exercise efficiency, allowing them to exercise at higher intensities for longer periods. Using music that is appropriate for the sport and preferred by the athlete is likely to enhance performance efficiency. Furthermore, the music in mood regulation plays a crucial role in enhancing athletes’ positive psychological capital. Hence, efforts and research are needed to develop and provide specific, practical strategies for applying this in sports settings to help athletes improve their positive psychological capital through music.

Second, it was found that music in mood regulation does not significantly impact athletic performance. This result was similar to findings that, although music positively affects mood, it does not significantly influence performance in situations that require high cognitive demands, such as driving (van der Zwaag et al., 2012). Although music in mood regulation helps athletes achieve a positive psychological state, as seen in Hypothesis 1, it may not have had a significant effect in this study because athletes must focus on various factors and competition situations when participating in events. However, the impact of music on athletic performance is likely to vary depending on the nature of the sport. For example, in sports that involve repetitive movements, such as marathon running, cycling, and swimming, music may enhance motivation and help reduce fatigue. In contrast, in sports that require high levels of concentration and strategic decision-making, such as gymnastics, golf, and fencing, music may act as a distraction rather than a performance aid (Bishop et al., 2008; Karageorghis and Terry, 2011). Furthermore, mood regulation through music may not function uniformly for all athletes, as individual personality traits (e.g., introversion vs. extraversion) and psychological states during competition can influence its effectiveness (Pates et al., 2003). Therefore, further research is needed to explore how music-based mood regulation interacts with athletes’ psychological characteristics and the specific demands of different sports disciplines. Specifically, an athlete’s performance is determined by a variety of factors, including physical fitness, skills, and psychology, making it difficult to expect significant results from music in mood regulation alone.

Contrary to it, the previous studies have shown that cellists manage emotions and achieve optimal performance through music (López-Íñiguez and McPherson, 2021), and that music improves performance and reduces fatigue (Li et al., 2022). This recommends that the relationship between music in mood regulation and athletic performance may vary across different fields and contexts. The lack of research directly analyzing the relationship between music in mood regulation and athletic performance makes it challenging to draw in-depth comparisons with previous studies. However, it could consider factors such as athletes’ personal characteristics, experiences, and musical preferences.

Athletes’ personal experiences and situations regarding music in mood regulation may differ. For some athletes, music in mood regulation might have a positive impact, while for others, it might not. For instance, if an athlete has had negative experiences where music in mood regulation adversely affected their psychological state, it could lead to increased stress or anxiety, hindering athletic performance. In a study Fang et al. (2020), it was found that listening to music decreased work performance, with stronger music having a more negative impact than lighter music. This indicates that the negative effects of music listening on performance outcomes may be interpreted similarly in this context. These findings suggest that music listening may not only serve as an emotional regulation tool but also increase cognitive load in certain contexts. Specifically, in situations where athletes must engage in strategic decision-making and rapid judgment during competition, music may divert cognitive resources and impair concentration. For example, listening to emotionally intense music in high-pressure competitive situations could interfere with an athlete’s attentional focus, potentially reducing performance efficiency (Bishop et al., 2008). Therefore, to gain a more in-depth understanding of the effects of music on athletic performance, future research should explore the interaction between music characteristics (e.g., tempo, genre) and athletes’ psychological states during competition.

In addition, the music in mood regulation factors used in this study—such as revival, discharge, strong sensation, entertainment, diversion, and solace—were composed of positive factors. This raises the possibility of question bias, as the measurement tool may not fully represent athletes’ situations or experiences. There is a need to analyze positive or negative experiences and perceptions of music in mood regulation using qualitative research methods, and to explore their relationship with athletic performance to recognize the limitations of quantitative research. Furthermore, to analyze the impact of music-based mood regulation on athletes’ psychological states and performance in more realistic contexts, it is necessary to incorporate both experimental studies and case studies. For example, conducting interviews and observational research on how athletes use music before and after competitions or during training could provide more empirical, data-driven insights (Terry and Karageorghis, 2006). Additionally, a longitudinal study tracking changes in psychological states before and after competitions, as well as the frequency and type of music used during athletic performance, would be valuable in identifying the long-term effects of music listening. Lastly, the effectiveness of music in mood regulation may vary depending on the study subject, field, specific situations, and con-textual factors.

Third, it was found that positive psychological capital significantly impacts athletic performance. This result was consistent with studies by Gabana (2019) and Lai et al. (2020), which reported that positive psychological capital plays a critical role in improving athletes’ performance and is directly linked to performance enhancement. The findings align with previous research (Jung and Song, 2024; Kang and Jang, 2022), which indicates that positive psycho-logical capital positively affects perceived performance. In other words, the positive psychological capital helps athletes trust in their performance capabilities, overcome various situations encountered during competitions, and achieve optimal outcomes based on hope for their goals.

The positive psychological capital in athletes is integrated through four components: self-efficacy, optimism, hope, and resilience. It below addresses these components individually. The self-efficacy refers to an individual’s belief in successfully performing the behaviors required to achieve desired outcomes in specific situations (Bandura, 1977). Athletes with high self-efficacy have strong beliefs in their abilities. Luthans et al. (2007a) reported that individuals with high self-efficacy set challenging goals for themselves and achieve success by striving to reach those goals. Thus, athletes with high self-efficacy are likely to experience less anxiety due to psychological stability, set challenging goals, and work towards achieving them, positively influencing their athletic performance.

Optimism refers to a disposition to have positive expectations for the future. Carver and Scheier (2002) stated that individuals with high optimism are more likely to recognize and improve their weaknesses and are motivated to improve and adapt to realistic environments. In other words, athletes are likely to attribute their abilities to successful out-comes, while failures in competitive situations are addressed through optimistic thinking, which improves athletic performance.

Hope involves a positive expectation and cognition that one can achieve specific goals. Luthans and Avolio (2014) stated that individuals with high hope actively create various pathways to achieve their goals. Accordingly, athletes’ hope induces constructive emotional responses, helping them set clear goals and find specific plans and creative solutions to achieve them, ultimately aiding their athletic performance.

Resilience is an adaptive resource that regulates emotions and alters situational and environmental contingencies, broadly encompassing flexible adaptability to internal and external stress (Rutter, 1987). It allows athletes to overcome stress and adversity, respond flexibly to unexpected situations in sports performance or competition, and maintain athletic performance. Athletes with high resilience are likely to have strong motivation and mental fortitude in facing risks or adversity, contributing positively to their athletic performance. This finding aligns with the idea that individuals with high positive psychological capital are better equipped to adapt when faced with negative experiences and environ-mental changes (Wang et al., 2014).

Finally, the positive psychological capital, which leverages athletes’ positive psychology and strengths, contributes to performance through constructive behaviors and thinking. The positive psychological capital can be developed and improved through learning and experience (Luthans et al., 2007a; Lim, 2014; Kim et al., 2017), indicating the need for program development and support to enhance the positive psychological capital.

## 5 Conclusion

The purpose of this study was to investigate the relationship between athletes’ music in mood regulation, positive psychological capital, and athletic performance. Based on the results, the following conclusions were drawn.

First, athletes’ music in mood regulation positively affects positive the psychological capital. Second, athletes’ music in mood regulation does not have a significant effect on the athletic performance. Third, athletes’ positive psychological capital positively affects the athletic performance. In conclusion, while athletes’ music in for mood regulation does not directly impact athletic performance, the positive psychology formed through music in mood regulation can positively influence the athletic performance. Therefore, it is necessary for coaches to focus on and support active education and strategies for music in mood regulation, which positively affects positive psychological capital.

The following suggestions were made for future research. This study focused on analyzing the importance and influence of the variable of music in mood regulation, which has not been extensively studied in the field of professional sports. Future research should analyze the sub-factors of music in mood regulation and their relationships with various variables and conduct studies that can elucidate specific processes and information. This would help in forming athletes’ positive psychological capital and ultimately improving their performance.

## Data Availability

The data that support the findings of this study are available from the corresponding author upon reasonable request.
